# Epidemiological Survey and Risk Factor Analysis of 14 Potential Pathogens in Golden Snub-Nosed Monkeys at Shennongjia National Nature Reserve, China

**DOI:** 10.3390/pathogens12030483

**Published:** 2023-03-18

**Authors:** Mingpu Qi, Qiankun Wang, Yu Wang, Yingyu Chen, Changmin Hu, Wanji Yang, Feng Wu, Tianpeng Huang, Ali Sobhy Dawood, Muhammad Zubair, Xiang Li, Jianguo Chen, Ian Duncan Robertson, Huanchun Chen, Aizhen Guo

**Affiliations:** 1 State Key Laboratory of Agricultural Microbiology, College of Veterinary Medicine, Hubei Hongshan Laboratory, Huazhong Agricultural University, Wuhan 430070, China; qimingpu@webmail.hzau.edu.cn (M.Q.); qiankun.wang@wustl.edu (Q.W.); y.wang22@massey.ac.nz (Y.W.); chenyingyu@mail.hzau.edu.cn (Y.C.); hcm@mail.hzau.edu.cn (C.H.); ali.dawood@vet.usc.edu.eg (A.S.D.); zubair_durani@webmail.hzau.edu.cn (M.Z.); chenjg@mail.hzau.edu.cn (J.C.); i.robertson@murdoch.edu.au (I.D.R.); chenhch@mail.hzau.edu.cn (H.C.); 2 EpiCentre, School of Veterinary Science, Massey University, Private Bag 11-222, Palmerston North 4442, New Zealand; 3 Hubei International Scientific and Technological Cooperation Base of Veterinary Epidemiology, The Cooperative Innovation Center for Sustainable Pig Production, Huazhong Agricultural University, Wuhan 430070, China; 4 National Professional Laboratory for Animal Tuberculosis (Wuhan) of Ministry of Agriculture and Rural Affairs, Huazhong Agricultural University, Wuhan 430070, China; 5 Key Laboratory of Development of Veterinary Diagnostic Products, Ministry of Agriculture of the People’s Republic of China, Wuhan 430070, China; 6 Key Laboratory of Conservation Biology for Shennongjia Golden Monkey, Shennongjia Forest District 442411, China; yangwanji8888@163.com (W.Y.); w13593715021@126.com (F.W.); htp1935677332@163.com (T.H.); 7 Infectious Diseases, Faculty of Veterinary Medicine, University of Sadat City, Sadat City 32897, Egypt; 8 Institute of Veterinary Medicine, Jiangsu Academy of Agricultural Sciences, Nanjing 210000, China; 9 College of Animal Science and Technology, Huazhong Agricultural University, Wuhan 430070, China; xxianglli@mail.hzau.edu.cn; 10 School of Veterinary Medicine, Murdoch University, Murdoch 6150, Australia

**Keywords:** golden snub-nosed monkey, epidemiology, tuberculosis, adenovirus, macacine herpesvirus 1, cytomegalovirus, simian foamy virus, hepatitis A virus

## Abstract

Golden snub-nosed monkeys (*Rhinopithecus roxellanae*) belong to Class A, the highest level of endangered primate species. Exploring the infection status of potential pathogens in golden snub-nosed monkeys is important for controlling associated diseases and protecting this species. The objective of this study was to investigate the seroprevalence for a number of potential pathogens and the prevalence of fecal adenovirus and rotavirus. A total of 283 fecal samples were collected from 100 golden snub-nosed monkeys in December 2014, June 2015, and January 2016; 26 blood samples were collected from 26 monkeys in June 2014, June 2015, January 2016 and November 2016 at Shennongjia National Reserve in Hubei, China. The infection of 11 potential viral diseases was examined serologically using an Indirect Enzyme-linked Immunosorbent Assay (iELISA) and Dot Immunobinding Assays (DIA), while the whole blood IFN-γ in vitro release assay was used to test tuberculosis (TB). In addition, fecal Adenovirus and Rotavirus were detected using Polymerase Chain Reaction (PCR). As a result, the Macacine herpesvirus-1 (MaHV-1), Golden snub-nosed monkey cytomegalovirus (GsmCMV), Simian foamy virus (SFV) and Hepatitis A virus (HAV) were detected with the seroprevalence of 57.7% (95% CI: 36.9, 76.6), 38.5% (95% CI: 20.2, 59.4), 26.9% (95% CI: 11.6, 47.8), and 7.7% (95% CI: 0.0, 84.2), respectively. Two fecal samples tested positive for Adenovirus (ADV) by PCR, with a prevalence of 0.7% (95% CI: 0.2, 2.5), and further, the amplification products were sequenced. Phylogenetic analysis revealed that they belonged to the HADV-G group. However, other pathogens, such as Coxsackievirus (CV), Measles virus (MeV), Rotavirus (RV), Simian immunodeficiency virus (SIV), Simian type D retroviruses (SRV), Simian-T-cell lymphotropic virus type 1 (STLV-1), Simian varicella virus (SVV), Simian virus 40 (SV40) and *Mycobacterium tuberculosis* complex (TB) were negative in all samples. In addition, a risk factor analysis indicated that the seroprevalence of MaHV-1 infection was significantly associated with old age (≥4 years). These results have important implications for understanding the health status and conservation of the endangered golden snub-nosed monkey population at Shennongjia Nature Reserve.

## 1. Introduction

Golden snub-nosed monkeys (*Rhinopithecus roxellanae*) belong to class A, the highest level of endangered species on the list of national protected animals in China [1]. They are also classified as an endangered species on the International Union for Conservation of Nature (IUCN) Red List [2]. There are about 25,000 monkeys, which are distributed in the Chinese provinces of Hubei, Sichuan, Gansu, and Shanxi [3,4,5]. In Hubei province, the monkeys mainly live in Shennongjia National Nature Reserve, where only 1471 monkeys were observed according to the third survey of golden snub-nosed monkeys conducted by the staff of Shennongjia Nature Reserve in 2018.

It is well known that animal diseases greatly impede animal conservation [6]. Further, with regard to the Shennongjia National Nature Reserve as an important eco-tourism area, a number of zoonotic pathogens in golden snub-nosed monkeys would potentially affect public health if shared with humans, such as *Mycobacterium tuberculosis* (*M.tb*), Simian virus 40 (SV40), Macacine herpesvirus-1 (MaHV-1, formerly called simian B Virus), Adenovirus, Rotavirus, Simian foamy virus (SFV), etc. [7,8,9]. TB is one of the major diseases of non-human primates (NHPs) and is caused mainly by *M.tb*, but also sometimes by *Mycobacterium bovis* (*M.bovis*) and other members of the *Mycobacterium tuberculosis complex* (MTBC) [7]. The mutual transmission of TB between NHPs and humans could occur when they are in close contact [10]. SV40 usually causes asymptomatic infection but can be activated when immunodeficiency occurs in monkeys [11]. The virus is thought to have been introduced into humans between 1955 and 1963 through contaminated polio vaccines. These vaccines were produced in SV40-infected monkeys and were circulated in human populations, becoming one of the most potent human carcinogens leading to the malignant transformation of human cells [12]. However, there is a possibility of cross-transmission between NHPs and humans [12]. MaHV-1 is an alpha-herpesvirus that is endemic to macaques and that causes localized self-limiting herpetic lesions, and then latency in sensory neural ganglia. Humans can be infected by monkey bites and scratches and develop fetal encephalitis with a mortality exceeding 70% in the absence of treatment and 20% even with adequate treatment [13]. SFV is ubiquitous in several primate species and usually causes persistent infection [14,15], whereas SFV infections in humans are well documented to cause subclinical hematologic changes [16,17,18]. In addition, Adenovirus and Rotavirus are important zoonotic pathogens that might lead to high rates of mortality in humans and animals [19,20,21]. In addition, some other primate pathogens, such as Rhesus cytomegalovirus (RhCMV) [22], Simian immunodeficiency virus (SIV) [23], Simian T-cell lymphotropic virus (STLV) [24], Simian type D retrovirus (SRV) [25], and Simian varicella virus (SVV) [26], cause clinical/subclinical diseases similar to the corresponding human pathogens, i.e., Human cytomegalovirus (HCMV) [27], Human immunodeficiency virus (HIV) [28], Human T-cell lymphotropic virus (HTLV) [24], Human type D retrovirus (HRV) [29] and Human varicella-zoster virus (VZV) [26], respectively. Although there is no English language research on SIV infecting Asian NHPs, a Chinese paper reported that 4.21% of 475 Chinese monkeys were seropositive to SIV [23]. 

Recently, there has been increasing focus on the population genetics [30,31], evolution [32] and behavior [33,34] of this species of monkeys. However, there are very few data describing the infection status of this species, likely due to difficulties sampling the endangered species. A few case studies based on opportunistic collections of carcasses from monkeys that have died for unknown reasons or on diseased captive monkeys in zoos have shown that golden snub-nosed monkeys could be infected with bacteria such as *Streptococcus* [35], *Staphylococcus* [36], *Shigella* [37] and *Escherichia coli* [38], Protozoan and Nematode parasites [39], and viruses such as Coxsackievirus B3 [40] and Adenovirus [41]. A better understanding of the health status of the Shennongjia golden snub-nosed monkeys is necessary to protect this endangered population more efficiently. Potential zoonotic pathogens in golden snub-nosed monkeys are a priority because of the potential risk of these pathogens being transmitted to the surrounding wild animals and humans in this famous AAAAA (the top rank) ecotourism Scenic Area in China. Based on findings regarding the pathogens present in golden snub-nosed monkeys and/or the other NHPs, and their zoonotic characteristics, 14 potential pathogens were selected. The aim of this study was to investigate the seroprevalence of a number of potential pathogens and their prevalence for fecal adenovirus and rotavirus in this species, and the association with risk-related factors (monkey’s age, sex, sampling time and growth pattern).

## 2. Materials and Methods

### 2.1. Ethics Statement

All animals involved were kept in strict accordance with the Guide for the Care and Use of Laboratory Animals, Hubei Province, China. The protocols were approved by the ethics committee of Huazhong Agricultural University (agreement no. HZAUMK-2014-001). 

### 2.2. Study Area and Monkey Information

**Figure 1:**
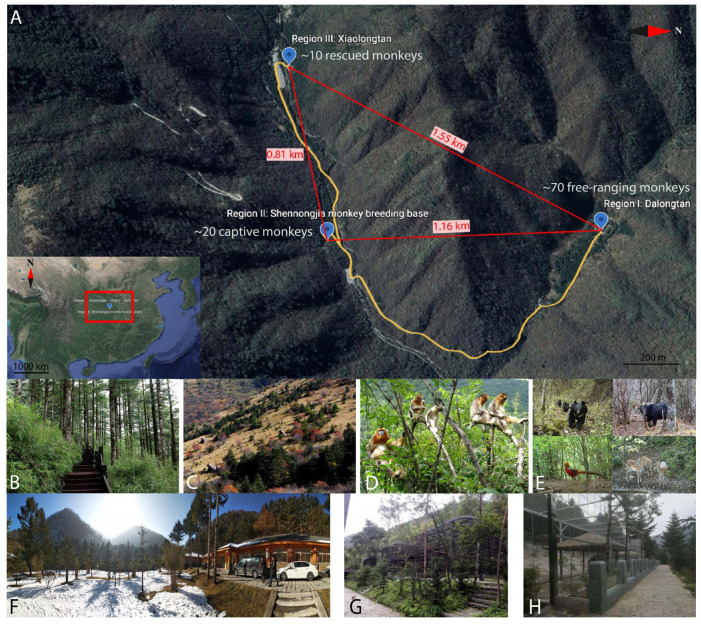
Distribution, living conditions and surrounding environment of the golden snub-nosed monkeys in Shennongjia National Nature Reserve, China. (**A**) Distribution of the monkeys. Region I (Dalongtan): the monkeys were free ranging; Region II (Xiaolongtan) and Region III (Shennongjia monkey breeding base): monkeys were fed in captivity. The three regions are connected by an asphalt road which is colored yellow, and the straight-line distance between the three regions is shown using red lines. (**B**–**E**) Surrounding environment of monkey habitat. (**B**,**C**): Vegetation coverage of Shennong Peak, (**D**): Monkeys prefer to stay in trees, (**E**): Wild animals found in Shennongjia. (**F**–**H**) Living conditions of monkeys. (**F**): Dalongtan; (**G**): Shennongjia monkey breeding base; (**H**): Xiaolongtan.

The study site was Shennong Peak, one of the AAAAA Scenic Areas in China, which is in Shennongjia National Nature Reserve. The Shennong Peak Scenic Area covers an area of about two square kilometers located at an altitude of 3106 meters (Figure 1A). This region has a subtropical monsoon climate, where the weather is cold, humid and changeable, with an average annual temperature of 7.9 °C. This region has some of the richest biodiversity in the country, where the vegetation is mainly composed of mixed coniferous and deciduous forests (Figure 1B,C) and the monkeys spend most of their time in the trees (Figure 1D); in addition, more than 700 vertebrates have also been found in this region, including the Asiatic black bear, the Naemorhedus caudatus, the sika deer and the Chrysolophus pictus (Figure 1E). Every year, more than 500,000 tourists visit Shennong Peak Scenic Area, where tourists can observe the monkeys in the wild from a distance and see captive monkeys up close.

About 100 golden snub-nosed monkeys were artificially (captive monkey) or semi-artificially (free-ranging monkey) kept in Shennongjia Nature Reserve in three regions, as follows. Region I: the Dalongtan area (110°31′93″ N, 31°49′25″ E), in which about 70 free-ranging monkeys lived, consisting of 4 one-male units (OMU) and 1 all-male unit (AMU) (Figure 1E); Region II: the Shennongjia breeding base for golden snub-nosed monkeys (110.31′74″ N 34, 31.48′59″ E), in which about 20 monkeys were kept, and where the monkeys were randomly divided into 3 OMUs by animal caretakers, with each unit caged in a large steel house (Figure 1F); Region III: the Xiaolongtan area (110°31′44″ N, 31°48′38″ E), which is used to hold rescued monkeys collected from different areas of Shennongjia Reserve with physical injuries (Figure 1G). There were about 10 monkeys in the cage, which were thus considered as one unit. The straight-line distance between each region was from 0.81 km to 1.55 km, but Region I is separated from region II and III by the mountain barrier. These three regions are connected by asphalt roads, which are easily accessible for researchers and tourists (Figure 1A).

All monkeys were fed sweet potatoes, oranges, peaches and apples three times a day (10:00–11:00; 14:00–15:00 and 18:00–19:00) depending on the season. The monkeys from region I usually came quickly from the wild to the trees at the feeding site at the fixed time after hearing the staff’s call. Then, they jumped down from the trees to get the food and ate it. After feeding, they immediately returned to the mountains to live in the wild. The monkeys in regions II and III, on the other hand, were kept in their cages and fed at a fixed time.

### 2.3. Sample Collection

In total, 283 fecal samples were collected in December 2014, June 2015, and January 2016 from all 100 monkeys following the protocol described in the previous study [38]. Briefly, a one-week field trip was performed for each sampling month, and fecal samples were collected by two researchers before the morning (10:00–11:00) and afternoon (14:00–15:00) feeding times, when the monkeys had already descended the mountain, congregated in the trees and were waiting for their food. Each researcher was responsible for one monkey unit per time. Fresh feces were collected into sterile disposable plastic bags immediately after the monkeys had defecated and stored in a portable 4 °C refrigerator. Each sampling session lasted approximately one hour, and the samples were returned to the laboratory and placed in a −20 °C freezer for further analysis. In addition, information about the approximate age and sex of the monkeys was also recorded during sampling as they stayed and jumped in the tall trees.

Considering the safety of monkeys in the context of the serious stress caused by blood collection, blood sampling was strictly limited by the local protective regulations for golden snub-nosed monkeys. A total of 26 clinically healthy and vigorous monkeys were selected for blood collection; further information on the monkeys from which blood was collected is listed in Table 1. The monkeys were anesthetized, and blood was collected from their hind limb veins after 12 h fasting in June 2014, June 2015, January 2016, and November 2016. The monkey’s heart and respiratory rates were carefully monitored by two experienced veterinarians throughout the period of blood collection until the monkeys recovered from anesthesia. In addition, sample information, including the monkey’s age (sub-adult monkey, aged less than 4 years old and adult monkey, aged 4 years or more), sex, growth patterns, and sampling time were documented as well. After the blood-taking process, the whole blood samples were stored in a heparinized tube at room temperature for the TB test with an IFN-γ in vitro release assay; the serum samples were separated by centrifugation at 2800 g for 10 min, collected into 1.5 ml microcentrifuge tubes and stored at −20 °C for antibody detection.

### 2.4. Interferon-γ (IFN-γ) In Vitro Release Assay to Test TB

The 26 heparinized whole blood samples were used for the TB test, which was performed by IFN-γ release assay, as described previously [42]. Briefly, bovine purified protein derivatives (bPPD) (1000 international units per milliliter [IU/mL], diluted into 50 IU/mL) (China Institute of Veterinary Drugs Control, Beijing, China), avian purified protein derivatives (aPPD) (1000 IU/mL, diluted into 50 IU/mL) (China Institute of Veterinary Drugs Control, Beijing, China), Concanavalin A (ConA) (5 mg/mL, diluted into 100 μg/mL) (Sigma, California, USA) were used as the positive control, and phosphate-buffered saline (PBS) (10X, diluted into 1X) (Takara, Dalian, China) was used as the negative control to stimulate the whole blood overnight. Then, the supernatant plasma was collected and the IFN-γ concentration was measured using the standard curve of the IFN-γ according to the instructions of the commercial human/monkey IFN-γ quantification kit (MABTECH, Stockholm, Sweden). Based on the criteria previously established by this laboratory, a sample was classified as TB positive if the difference in the IFN-γ concentration between the bPPD- and aPPD-stimulated samples was equal to or greater than 114.5 pg/mL [43].

### 2.5. Detection of Serum Antibodies to 11 Viruses

In total, 26 serum samples were used for the virus antibody test. Commercial indirect Enzyme-linked Immunosorbent Assay (iELISA) kits for the detection of CV (SERION ELISA classic Coxsackievirus B3 IgG), MeV (SERION ELISA classic Measles Virus IgG), and GsmCMV (SERION ELISA classic Rhesus Monkey Cytomegalovirus IgG) were purchased from VIRION–SERION (Würzburg, Germany). HAV (Hepatitis A IgG antibody detection kit) and SVV (Simian varicella virus antibody detection kit) were detected using indirect ELISA kits from VRL (Suzhou, China). All procedures were performed according to the manufacturer’s protocol. Briefly, serum samples were heat-inactivated before assays. Then, they were added into the wells and incubated with the coated antigens in the ELISA plates for 60 min at 37 °C; then, the conjugated secondary antibodies were added to the plates and incubated at 37 °C for 30 minutes, which was followed by the addition of the p-nitrophenyl phosphate (pNPP) substrate solution and further incubation at 25 °C for 30 min. Finally, the reaction was stopped, and the absorbance in each well was measured at 405 nm. 

MaHV-1 (Monkey B virus dot immunoassay kit), SFV (Simian Foamy virus dot immunoassay kit), SIV (Simian Immunodeficiency virus dot immunoassay kit), SRV (Simian retrovirus type D dot immunoassay kit), STLV–1 (Simian T-cell leukemia virus type 1 dot immunoassay kit), and SV40 (Simian virus 40 dot immunoassay kit) were detected using commercial dot immunobinding assay (DIA) kits (VRL, Suzhou, China). Briefly, the antigen-coated membrane was soaked in blocking buffer (5% non-fat dry milk) and then samples were added with the filter paper strip, which was incubated at 37 °C for 30 min; this was followed by the incubation of the secondary antibody, which was conjugated with alkaline phosphatase (ALP), and the positive signal was developed by 5-bromo-4-chloro-3-indolyl-phosphate/Nitro-Blue-Tetrazolium (BCIP/NBT). Finally, both the positive and negative results of the samples were determined visually by color intensity compared with the positive and negative controls. 

### 2.6. PCR and Phylogenetic Analysis of Adenovirus and Rotavirus

DNA and RNA were extracted from all fecal samples by using a fecal nucleic acid extraction kit (ZD Biotech, Ningbo, China). The cDNA synthesis was performed using the PrimeScript reagent kit with gDNA Eraser (Takara, Dalian, China). All procedures were performed according to the manufacturer’s protocols. The extracted DNA and synthesized cDNA were subjected to PCR amplification. Specific primers of two enteric viruses, Adenovirus and Rotavirus, were designed and synthesized by Sangon Biological Engineering Technology and Service Co., Ltd. (Shanghai, China) (Table 2). The amplification was performed using the 2×Taq Plus Master Mix (Vazyme, Nanjing, China). The total volume of the reaction was 25 μL, containing 12.5 μL of 2×Taq Plus Master Mix, 1 μL of nucleic acid, 1 μL of 10mM forward primer, 1 μL of 10 mM reverse primer, and 9.5 μL of ddH_2_O, with a pre-denaturation step of 5 min at 94 °C; this was followed by 30 thermal cycles of denaturation at 94 °C for 30 s, the annealing temperature (Table 2) for 30 s, the extension at 72 °C for 1 min, and a final single extension step at 72 °C for 5 min. Amplicons were sequenced by Sangon Biological Engineering Technology and Service Co., Ltd. (Shanghai, China).

All the available sequences of the human adenovirus (HADV) A- G and simian adenovirus (SADV) A- I strains were retrieved from GenBank and aligned with the 2 sequences from this study using Multiple Alignment with a Fast Fourier Transform (MAFFT) [46] in Geneious Prime 2019. Preliminary Maximum Likelihood (PHYML) phylogenetic trees were generated in Geneious Prime 2019, using a General Time Reversible (GTR) substitution model. Sequences without an evolutionary relationship with the 2 sequences in this study were removed, leaving the representative sequences of 58 HADV and 9 SADV for further evolutionary analysis. The total 69 sequences were realigned using MAFFT in Geneious Prime 2019 in order to calculate the nucleotide and amino acid sequence identity. Final PHYML phylogenetic trees were bootstrapped, with 1000 replicates [47].

### 2.7. Statistical Analysis

Univariable and multivariable logistic regression models were developed to identify the potential risk factors associated with the above pathogens. The outcome variable is the serological test results, and the predictor variables include age (adult and sub-adult), sex (male and female), growth pattern (captive and free-ranging) and sampling season (summer and winter). Variables with a *p* value ≤ 0.20 in the univariable analyses were first subjected to a saturated multivariable logistic regression model. The model was refined using a stepwise back–forward process, and the best fitting model was determined using the likelihood ratio test and the minimum Akaike Information Criterion (AIC) value. The confounder factors were retained in the final model if their removal caused the odds ratio of other variables to change by more than 20%. The model was further assessed with the Hosmer–Lemeshow goodness-of-fit test and a receiver operating characteristic analysis. The odds ratio (OR) and its 95% confidence intervals (95% CIs) were also calculated to estimate the degree of association between different variables and seropositivity for each pathogen. Furthermore, we also examined the degree of association between the ≥1 seropositivity against the 14 tested pathogens and the possible variables, as described above. All data analysis and plots were performed using the R 4.1.2 [48] with the ‘car’ [49] package.

## 3. Results

### 3.1. Sample Information

In total, 283 fecal samples were collected, 49 from about 30 captive monkeys and 234 from about 70 free-ranging monkeys. Because we cannot accurately identify individual monkeys when they are far away from us, the repeated samples cannot be attributed to an individual monkey.

The 26 serum samples were collected from 26 individual monkeys without replicates. The 26 monkeys were divided into groups according to their age, sex, growth pattern, and sampling season. In general, 8 monkeys were sub-adults and 18 were adults; 11 were males and 15 were females, 13 monkeys were bred in captivity and 13 lived freely in the wild, and 20 blood samples were collected in summer (June) and 6 were collected in winter (January and November) (Table 1). 

### 3.2. Prevalence of the Investigated Pathogens at Sampling Periods

The overall seroprevalence of MaHV-1, GsmCMV, SFV and HAV was 57.7% (95% CI: 36.9, 76.6), 38.5% (95% CI: 20.2, 59.4), 26.9% (95% CI: 11.6, 47.8) and 7.7% (95% CI: 0.0, 84.2), respectively. MaHV-1 antibody prevalence remained at a high level in both winters (50.0%, in Jan. 2016, and Nov. 2016) and summer (60.0% in Jun. 2014 and Jun. 2015), indicating that MaHV-1 is a predominant pathogen carried in the monkey population. GsmCMV was also at a high level compared to the average seroprevalence of 20.0% in June 2014; it increased to 50.0% in Jun. 2015, Jan. 2016 and Nov. 2016. In addition, GsmCMV showed an increasing trend from Jan. 2014 to June 2015 and remained at a high level thereafter. SFV and HAV also showed an increasing prevalence, even to an extremely high level of 100% (95% CI: 15.8, 100). 

For ADV, it was only detected in the first sampling time with a prevalence of 2.0% (95% CI: 0.6, 7.1), and the overall prevalence of ADV was 0.7% (95% CI: 0.2, 2.5). 

Other pathogens were negative in either antibody tests or PCR tests (Table 3 and Table 4).

Furthermore, of those 26 monkeys, only 26.9% (95% CI: 11.6, 47.8) tested negative to all pathogens, 34.6% (95% CI: 17.2, 55.7) were infected with only one virus, and 38.5% (95% CI: 20.2, 59.4) had co-infection with two or three viruses (Table S1). 

### 3.3. Genetic and Phylogenetic Analysis of Adenovirus

**Figure 2:**
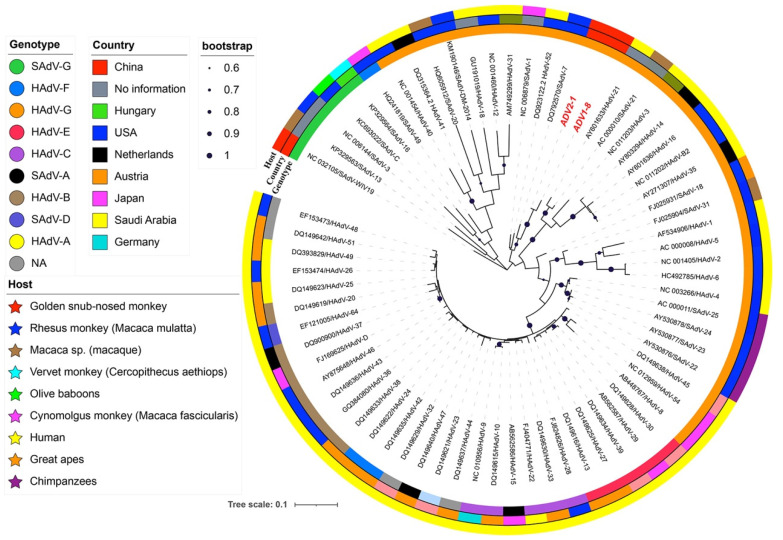
Maximum likelihood phylogenetic tree of the adenovirus strains in the golden snub-nosed monkeys at Shennongjia National Nature Reserve, China. Sequence names from this study were colored in red bold. The three outer rings, from inside to outside, are genotypes of the sequences, original countries of the strains, and the hosts of the strains; bootstrap values > 60% were shown.

Of the 283 fecal samples, two monkeys were positive for ADV, and the amplification products of the partial H gene were sequenced (GenBank accession numbers: OM674686 and OM674687). Phylogenetic relationships showed that both strains belonged to HADV group G and were clustered with SAdV-1, SadV-7, and HadV-52 (Figure 2), each sharing a 97.4% nucleotide (nt) and 100.0% amino acid (aa) identity with each other, and sharing a 75.5–92.1% nt and 89.4–100% aa identity with the other 67 adenoviruses, respectively. 

### 3.4. Risk Factor Analysis

The results of the univariable analysis are summarized in Table 5. In the final multivariable logistic model, only the adult age was significantly associated with MaHV-1 seropositivity at the animal level (OR = 33.0, 95% CI: 2.5, 443.6, *p* = 0.002), and the sampling in summer had a slightly higher probability of being MaHV-1 seropositive (OR = 5.5, 95% CI: 0.6, 49.5, *p* = 0.128). For GsmCMV, only the adult age group had a slightly higher GsmCMV seropositivity with an OR of 5.4 (95% CI: 0.5, 53.9, *p* = 0.106), compared to the sub-adult age. Female golden snub-nosed monkeys were slightly more likely to be SFV seropositive than the male monkeys (OR = 5.4, 95% CI: 0.5, 53.9, *p* = 0.106) (Table 6). However, when all diseases were considered together (at least 1 seropositive against one pathogen), only adult monkeys were significantly more likely to be seropositive to ≥1 pathogen tested, compared to sub-adult monkeys (OR = 21.3, 95% CI: 2.4, 191.6, *p* = 0.003) (Table 7).

## 4. Discussion

The golden snub-nosed monkey is one of the most well-known endangered primates in the world. Due to its small population in only a few natural habitats, there is an urgent need for dynamic protection [50]. It is important to establish a data archive of infectious diseases for protecting these endangered animals. Recently, there has been an increase in the quantity of research on golden snub-nosed monkeys, with the major focus areas including genome sequencing and genetic evolution, origin, population history, and behaviors such as sleeping sites, tree selection, and routine allomaternal nursing [30,31,32,33,34]; however, an epidemiological investigation into the multiple pathogens that infect and potentially threaten golden snub-nosed monkeys has been rarely reported. This study sampled the monkeys four times over a three-year period and screened the infections caused by fourteen potential pathogens that pose a potential threat to public health, mainly by using fecal samples and some blood samples collected during the summer and winter seasons. As a result, the infection of five pathogens, including MaHV-1, GsmCMV, SFV, HAV, and ADV, was detected. The seroprevalence of the first four pathogens was at a high level, and GsmCMV, SFV and HAV showed an increasing trend with sampling year. Considering age as a risk factor for the investigated diseases, only BV prevalence showed a significant association with age. Further, the proportion of co-infection was as high as 38.5% (95% CI: 20.2, 59.4). Regarding the potential threat to animals and humans due to the three zoonotic pathogens MaHV-1, HAV and ADV, regular surveillance is necessary and strongly recommended because of the difficult and strictly restricted blood sampling of this endangered species.

MaHV-1 is commonly responsible for either asymptotic or mild disease in monkeys, but it can also lead to severe brain damage (such as fetal encephalitic infection) or death in humans [51]. Human zoonotic cases of MaHV-1 have been reported in China and other countries. At least 50 people have been infected and 21 of them died [52]. The infected humans were veterinarians, monkey care personnel, or laboratory workers conducting research on monkeys; for example, the Chinese case involved a veterinary surgeon who dissected two dead monkeys and was infected with MaHV-1 [52]. In this study, MaHV-1 had an overall real seroprevalence of 57.7% (95% CI: 36.9, 76.6), which is similar to the 52.6% MaHV-1 seroprevalence in golden snub-nosed monkey reported in 1993 [53]; it is slightly higher than the seroprevalence of MaHV-1 reported in long-tailed macaques (39%) [54] and rhesus macaques (25.8% in the USA and 45% in Brazil) [55,56]. Previous studies also reported that the seroprevalence of MaHV-1 tends to increase after capturing the animals and caging them in dense groups [57]. A significant positive association existed between the monkey’s age (≥4 years) and the seroprevalence of MaHV-1 (OR = 33.0, 95% CI: 2.5, 443.6), which is consistent with previous findings [54,55,56]. Therefore, the high prevalence of MaHV-1 in the golden snub-nosed monkey population may be attributed partially to the higher proportion of adults in the sampled monkeys (19 adult monkeys vs. 7 sub-adult monkeys). 

HAV is an emerging public health concern and is responsible for over 1.5 million annual cases in humans globally [58]. Concerning the host range of HAV, it is limited to humans and several species of NHPs [59,60]. However, several NHPs tested positive for HAV and a few clinical signs of HAV-infected NHPs were reported. The literature has documented the outbreak of spontaneous hepatitis A in imported rhesus monkeys showing clinical signs similar to those in humans [61]. To the best of our knowledge, there is no previous research on HAV infection in golden snub-nosed monkeys. Moreover, the prevalence level is consistent with a previous study suggesting that HAV infection existed in several captive and wild NHPs, with several rates of prevalence evident in the great apes (chimpanzee), Old World (cynomolgus, African vervet, stump-tailed) and New World monkeys (Aotus). Despite a significant degree of genomic heterogeneity in simian HAV isolates, they are considered to be a single serotype [59,62]. Regarding golden snub-nosed monkeys as a potential source of HAV, evidence from the current literature indicates that NHPs could be infected by HAV through close contact with infected humans/NHPs [63]. In addition, contaminated fruits and vegetables are common conduits for HAV infection in humans [64,65]. However, the particular reasons for golden snub-nosed monkeys being infected with HAV needs to be investigated further.

ADV is responsible for various diseases, including respiratory infection, gastroenteric infection, and hepatitis in NHPs and humans [41,66]. The prevalence of ADV-associated diseases in NHPs is associated with their health status [67,68]. According to the limited documents, ADV prevalence in diarrheal primates is 46% [67], while in the asymptomatic monkeys, it is 6.5% and 7% [67,68]. In this study, fecal samples were collected from clinically healthy monkeys three times in 2014, 2015, and 2016, and the occurrence of ADV was examined by PCR. ADV was detected only in samples collected in December 2014, showing prevalence of 2.0%. The value of the prevalence is close to the prevalence of ADV (5.1%) in golden snub-nosed monkeys reported by Tan [41]. Phylogenetic analysis indicated that two ADV strains in the current study were clustered into HADV-G, which was different from the WIV19 Tan strain, previously identified and clustered into SADV-G (Figure 2). Therefore, at least two genotypes of ADV were supposed to be circulating in the Shennongjia golden snub-nosed monkey population.

CMV is known as one of the best host-adapted opportunistic pathogens, and is characterized by slow replication, the establishment of latency, and frequent reactivation [69,70]. CMV infections in humans and NHPs are usually asymptomatic [71, but 15% of the infants, who are congenitally infected with CMV, develop neurological sequelae, which primarily manifest as progressive hearing loss [71]. On the other hand, CMV can also cause a severe disease in fetal macaques, which is similar to the pathogenesis of human CMV [71,72]. Humans and a wide range of NHPs are susceptible to CMV [70,73]. CMV prevalence in rhesus monkeys was close to 100% in some countries such as USA and Brazil [56,74]. CMV is a species-specific virus, but CMV antiserum has cross-reactivity among various CMV species [70]. In this study, the average GsmCMV seroprevalence in golden snub-nosed monkeys reached up to 38.3% (95% CI: 19.6, 59.6), as detected by using the RhCMV antigen, and is slightly lower than that in other primates [56,74]. The prevalence of GsmCMV might be affected by the possible existence of heterology between RhCMV and GsmCMV antigens [75], and the limited number of blood samples.

SFV is a ubiquitous retrovirus that causes persistent infections in NHPs and is transmitted across the monkeys via fights and bites [76]. In this study, SFV was tested positive for in June 2015 and reached up to 100% (95% CI: 14.0, 100) prevalence in November 2016 in Shennongjia Nature Reserve, which is similar to the previously reported SFV seroprevalence of 75–100% in adult macaques [77,78]. In addition, SFV could be transmitted to humans occupationally exposed to nonhuman primates, or who have contact with these animals in the same natural settings through the bites and scratches of African green monkeys, baboons and a variety of other NHPs, resulting in subclinical hematologic alterations in humans [14]. Thus, it poses a public health concern.

Based on the severity of the diseases in monkeys and of zoonotic diseases in humans, among the 5 pathogens detected in this study, ADV and HAV pose a higher risk to golden snub-nosed monkeys, as these two viruses could cause acute diseases; meanwhile, based on the current research, the risk of MaHV-1, GsmCMV and SFV in monkeys is lower. In addition, ADV, MaHV-1 and HAV pose the highest risk to humans, while the risk of CMV and SFV is lower [13,14,20,66,69]. This is because Shennongjia is one of the most famous tourist destinations in China and visitors to Shennongjia might have close contact with the monkeys by feeding them, playing with them, and walking around. Pathogens can be transmitted via direct contact, bites, scratches or contaminated water. In addition, workers in Shennongjia are more likely to be infected due to direct contact. Therefore, it is necessary to have knowledge about the possible transmission of these zoonotic pathogens between monkeys and humans. 

However, there are some limitations in this work. Firstly, the collection of blood samples was very limited since these monkeys belong to class A endangered species and taking blood from a large number of monkeys is not permitted. An inadequate number of blood samples results in a larger confidence interval, which affects the precision of the prevalence; in addition, some correlations (Odds ratio) between pathogens and risk factors cannot be calculated due to the smaller sample size. Secondly, although we attempted to use many more fecal samples to compensate for the disadvantage in terms of blood sampling, it is possible that there exist some gaps in the detection of infection by various pathogens between the blood and fecal samples that were collected by the noninvasive sampling method [79]. For instance, animals may be shedding pathogens intermittently, so serostatus may not match with the shedding status; in addition, past infections could result in persistent antibodies, but would no longer result in shedding. Thirdly, during the sampling period, we did not meet sick monkeys. Therefore, the monkeys from whom we collected the samples were all clinically healthy; thus, the corresponding data might underestimate the prevalence of the tested pathogens. Lastly, for fecal sampling, the age and sex of the monkeys could not be determined exactly, creating limitations in adjusting the data for further a risk factor analysis.

Many unknown aspects related to our current findings ought to be further investigated, such as the variance in prevalence with time, the possible increased morbidity with the same pathogens in the presence of some environmental stressor(s), and the impact of the mentioned infections on the status of the healthy monkeys at different stages of infection and new pathogens. The diseased monkeys are usually hidden in the forest and so are not available for sampling. Therefore, in the current study, we could not detect the real health status of the animals when initially infected. In the future, cooperation with the rangers and inspectors in the rescue stations should be strengthened in order to find the diseased monkeys and diagnose the related diseases in time. More physiological parameters should be detected for these clinically healthy monkeys to explore the subclinical abnormal status caused by the mentioned infections that could affect their survival capabilities in adverse environments, their reproduction performances and growth and longevity.

In conclusion, this study investigated the seroprevalence of twelve potential pathogens and the presence of fecal ADV and Rotavirus in Shennongjia golden snub-nosed monkeys and found the prevalence of five pathogen infections. Related risk factors were analyzed, and further recommendations were proposed in terms of the long-term monitoring of these pathogens in the monkey population, especially ADV, MaHV-1 and HAV infection in the adults (≥4 years), for the protection of this endangered species and for public health. Therefore, this study is of significance in the protection of this rare species of monkeys.

## Figures and Tables

**Table 1:** Individual information of blood samples taken from golden snub-nosed monkeys (*Rhinopithecus roxellanae*) (n = 26) at Shennongjia National Nature Reserve, China.

Monkey NO.	Sampling Time	Growth Patterns	Sex	Age *
1	June 2014	Captive	M	Sub-adult
2	June 2014	Captive	M	Sub-adult
3	June 2014	Captive	M	Adult
4	June 2014	Captive	M	Adult
5	June 2014	Captive	M	Adult
6	June 2014	Captive	M	Adult
7	June 2014	Captive	F	Sub-adult
8	June 2014	Captive	F	Sub-adult
9	June 2014	Captive	F	Sub-adult
10	June 2014	Captive	F	Adult
11	June 2015	Captive	F	Adult
12	June 2015	Captive	F	Adult
13	June 2015	Captive	F	Adult
14	June 2015	Free ranging	M	Adult
15	June 2015	Free ranging	M	Adult
16	June 2015	Free ranging	M	Adult
17	June 2015	Free ranging	M	Adult
18	June 2015	Free ranging	M	Adult
19	June 2015	Free ranging	F	Sub-adult
20	June 2015	Free ranging	F	Sub-adult
21	January 2016	Free ranging	F	Sub-adult
22	January 2016	Free ranging	F	Adult
23	January 2016	Free ranging	F	Adult
24	January 2016	Free ranging	F	Adult
25	November 2016	Free ranging	F	Adult
26	November 2016	Free ranging	F	Adult

* Sub-adult monkeys: <4 years old age; Adult monkeys: ≥4 years old age.

**Table 2:** Primer information for amplification of adenovirus and rotavirus in fecal samples from golden snub-nosed monkeys (*Rhinopithecus roxellanae*) at Shennongjia National Nature Reserve, China.

Target Viruses	Target Genes	Sequence (5’ to 3’)	Annealing Temperature	Length of Products	References
Adenovirus	hexon	TTCCCCATGGCICAYAACAC	50 °C	453 bp	[44]
		CCCTGGTAKCCRATRTTGTA			
Rotavirus	VP7	AAAGGATGGCCAACAGGATCAGT	52 °C	569 bp	[45]
		GTATARAAHACTTGCCACCAT			

**Table 3:** Seroprevalence of infection by *Mycobacterium tuberculosis* and 11 viruses in golden snub-nosed monkeys at Shennongjia National Nature Reserve, China.

	Jun. 2014 (n = 10)	Jun. 2015 (n = 10)	Jan. 2016 (n = 4)	Nov. 2016 (n = 2)	Total (n = 26)
Pathogens	TP% (95% CI: lower, upper)	TP% (95% CI: lower, upper)	TP% (95% CI: lower, upper)	TP% (95% CI: lower, upper)	TP% (95% CI: lower, upper)
MaHV-1	60.0 (26.2, 87.8)	60.0 (26.2, 87.8)	50.0 (6.8, 93.2)	50.0 (1.3, 98.7)	57.7 (36.9,76.6)
CV	0.0 (0.0, 30.8)	0.0 (0.0, 30.8)	0.0 (0.0, 60.2)	0.0 (0.0, 84.2)	0.0 (0.0, 13.2)
HAV	0.0 (0.0, 30.8)	0.0 (0.0, 30.8)	0.0 (0.0, 60.2)	100.0 (15.8, 100)	7.7 (0.0, 84.2)
Measles	0.0 (0.0, 30.8)	0.0 (0.0, 30.8)	0.0 (0.0, 60.2)	0.0 (0.0, 84.2)	0.0 (0.0, 13.2)
*M.tb*	0.0 (0.0, 30.8)	0.0 (0.0, 30.8)	0.0 (0.0, 60.2)	0.0 (0.0, 84.2)	0.0 (0.0, 13.2)
GsmCMV	20.0 (2.5, 55.6)	50.0 (6.8, 93.2)	50.0 (6.8, 93.2)	50.0 (1.3, 98.7)	38.5 (20.2, 59.4)
SFV	0.0 (0.0, 30.8)	40.0 (12.2, 73.8)	25.0 (0.6, 80.7)	100.0 (15.8, 100)	26.9 (11.6, 47.8)
SIV	0.0 (0.0, 30.8)	0.0 (0.0, 30.8)	0.0 (0.0, 60.2)	0.0 (0.0, 84.2)	0.0 (0.0, 13.2)
SRV	0.0 (0.0, 30.8)	0.0 (0.0, 30.8)	0.0 (0.0, 60.2)	0.0 (0.0, 84.2)	0.0 (0.0, 13.2)
STLV-1	0.0 (0.0, 30.8)	0.0 (0.0, 30.8)	0.0 (0.0, 60.2)	0.0 (0.0, 84.2)	0.0 (0.0, 13.2)
SV40	0.0 (0.0, 30.8)	0.0 (0.0, 30.8)	0.0 (0.0, 60.2)	0.0 (0.0, 84.2)	0.0 (0.0, 13.2)
SVV	0.0 (0.0, 30.8)	0.0 (0.0, 30.8)	0.0 (0.0, 60.2)	0.0 (0.0, 84.2)	0.0 (0.0, 13.2)

TP: Test prevalence, CI: confidence interval, MaHV-1: Macacine herpesvirus-1, CV: Coxsackievirus, HAV: Hepatitis A virus, *M.tb*: *Mycobacterium tuberculosis*, GsmCMV: Golden snub-nosed monkey cytomegalovirus, SFV: Simian Foamy virus, SIV: Simian immunodeficiency virus, SRV: Simian type D retrovirus, STLV-1: Simian T-cell lymphotropic virus type 1, SV40: Simian virus 40, SVV: Simian varicella virus.

**Table 4:** Test prevalence of fecal Adenovirus and Rotavirus detected with PCR in golden snub-nosed monkeys at Shennongjia National Nature Reserve, China.

	Dec. 2014	Jun. 2015	Jan. 2016	Total
Pathogens	% (95% CI: lower, upper)	% (95% CI: lower, upper)	% (95% CI: lower, upper)	% (95% CI: lower, upper)
Adenovirus	2.0 (0.6, 7.1)	0 (0, 4.1)	0 (0, 3.9)	0.7 (0.2, 2.5)
Rotavirus	0 (0, 3.8)	0 (0, 4.1)	0 (0, 3.9)	0 (0, 0.13)

TP: Test prevalence; CI: Confidence interval.

**Table 5:** Univariable analysis of risk factors (age, sex, growth pattern and season) associated with Macacine herpesvirus-1, Golden snub-nosed monkey cytomegalovirus and Simian Foamy virus in golden snub-nosed monkeys at Shennongjia National Nature Reserve, China.

Variables		MaHV-1			GsmCMV			SFV		
		Crude OR	95% CI	*p* value	Crude OR	95% CI	*p* value	Crude OR	95% CI	*p* value
Age	Adult vs. Sub-adult	16.8	1.6, 176.2	0.005	5.4	0.5, 53.9	0.106	-	-	-
Sex	Male vs. Female	0.9	0.2, 4.3	0.850	0.9	0.2, 4.6	0.899	5.4	0.5, 53.9	0.106
Growth patterns	Captive vs. Free-ranging	0.4	0.1, 1.9	0.231	1.0	0.2, 4.9	1.0	1.5	0.3, 8.5	0.658
Seasons	Summer vs. Winter	1.5	0.2, 9.4	0.665	0.5	0.1, 3.4	0.512	0.3	0.0, 1.7	0.162

OR: Odds Ratio, CI: Confidence interval, MaHV-1: Macacine herpesvirus-1, GsmCMV: Golden snub-nosed monkey cytomegalovirus, SFV: Simian Foamy virus, -: not statistically significant.

**Table 6:** Multivariable logistic regression analysis of risk factors (age, sex and season) associated with Macacine herpesvirus-1, Golden snub-nosed monkey cytomegalovirus and Simian Foamy virus seropositivity in golden snub-nosed monkeys at Shennongjia National Nature Reserve, China.

Variables		MaHV-1			GsmCMV			SFV		
		Adjusted OR	95% CI	*p* value	Adjusted OR	95% CI	*p* value	Adjusted OR	95% CI	*p* value
Age	Adult vs. Sub-adult	33.0	2.5, 443.6	0.002	5.4	0.5, 53.9	0.106	-	-	-
Sex	Male vs. Female	-	-	-	-	-	-	5.4	0.5, 53.9	0.106
Seasons	Summer vs. Winter	5.5	0.6, 49.5	0.128	-	-	-	-	-	-

OR: Odds ratio; CI: Confidence interval, MaHV-1: Macacine herpesvirus-1, GsmCMV: Golden snub-nosed monkey cytomegalovirus, SFV: Simian Foamy virus, -: not statistically significant.

**Table 7:** Univariable and multivariable analysis of risk factors (age, sex, growth pattern and season) associated with monkeys infected with pathogens and monkeys without infection.

Variables	Category	Crude OR (95% CI)	*p* Value	Adjusted OR (95% CI)	*p* Value
Sex	Female vs. Male	0.6 (0.1, 3.6)	0.524	-	-
Age	Adult vs. Sub-adult	21.3 (2.4, 191.6)	0.003	21.3 (2.4, 191.6)	0.003
Growth pattern	Captive vs. Free-ranging	0.3 (0.04, 1.9)	0.179	-	-
Season	Summer vs. Winter	0.5 (0.04, 4.9)	0.503	-	-

OR: odds ratio; CI: confidence interval; -: not statistically significant.

## Data Availability

All of the sequences of ADV were deposited in GenBank.

## Supplementary Materials

The following supporting information can be downloaded at: https://www.mdpi.com/article/10.3390/pathogens12030483/s1, Table S1: Information on the growth pattern, sampling season, age, sex and infection status of golden snub-nosed monkeys (n = 26) at Shennongjia National Nature Reserve, China.
