# Supplementary material for: Epidemiological Survey and Risk Factor Analysis of 14 Potential Pathogens in Golden Snub-Nosed Monkeys at Shennongjia National Nature Reserve, China
Source: Pathogens. 2023 Mar 18;12(3):483. doi: 10.3390/pathogens12030483 (PMC10051804; doi:10.3390/pathogens12030483)
Supplement: Supplementary file 1 [file pathogens-12-00483-s001.zip › pathogens-2252770-supplementary.pdf]

Supplementary Table S1. Information on the growth pattern, sampling season, age, sex and infection status of golden snub-nosed monkeys (n=26) at Shennongjia National Nature Reserve, China.

Monkey name	Raising patterns	Seasons	Age	Sex	MAHV-1	HAV	RhCMV	SFV	Infection status
Jiaojiao	C	S	Adult	F	×	×	×	×	No infection
Beibei	C	S	Adult	M	×	×	×	×	No infection
Huahua	C	S	Adult	F	√	×	×	×	Single infection
Qiangqiang	C	S	Adult	M	√	×	√	×	Double infection
Shennv	C	S	Sub-adult	F	√	×	×	×	Single infection
Linlin	C	S	Sub-adult	F	×	×	×	×	No infection
Qiaoqiao	C	S	Adult	F	×	×	×	×	No infection
Xiaohongtou	F	S	Adult	M	√	×	√	×	Double infection
Xingxing	F	S	Adult	M	√	×	×	×	Single infection
Changmao	F	S	Adult	M	√	×	×	×	Single infection
Kaixin	F	S	Adult	F	×	×	×	√	Single infection
Tuanyuan	F	S	Sub-adult	F	√	×	×	×	Single infection
Yezi	F	S	Sub-adult	F	×	×	×	×	No infection
Xiaoli	F	S	Adult	F	√	×	√	×	Double infection
Xingfeng	F	S	Adult	F	√	×	×	√	Double infection
Xiaolongnv	F	S	Sub-adult	F	√	×	√	×	Double infection
Duanwei	C	S	Adult	M	√	×	√	√	Triple infection
Fanfan	C	S	Sub-adult	M	×	×	√	×	Single infection
Dahai	C	S	Sub-adult	M	×	×	×	×	No infection
Junjun	F	S	Adult	F	√	×	√	√	Triple infection
21	F	W	Adult	F	×	×	×	×	No infection
22	F	W	Adult	F	√	×	√	√	Triple infection
23	F	W	Adult	M	√	×	×	×	Single infection
24	F	W	Adult	M	×	×	√	×	Single infection
25	C	W	Adult	F	√	√	√	√	Quadruple infection
26	C	W	Adult	F	×	√	×	√	Double infection

Raising patterns:(C: Captive, F: Free-ranging, S: Summer, W: Winter; Sex: F: Female, M: Male; ×: not infected; √: infected.
